# The dynamic uptake and release of SOD3 from intracellular stores in macrophages modulates the inflammatory response

**DOI:** 10.1016/j.redox.2019.101268

**Published:** 2019-07-02

**Authors:** Lili Hu, Elias D. Zachariae, Ulrike G. Larsen, Frederik Vilhardt, Steen V. Petersen

**Affiliations:** a Department of Biomedicine, Aarhus University, DK-8000, Aarhus C, Denmark; b Department of Cellular and Molecular Medicine, University of Copenhagen, DK-2200, Copenhagen N, Denmark

**Keywords:** Superoxide dismutase 3 (SOD3), Macrophage, Internalization, Secretion, Extracellular redox regulation, LRP1

## Abstract

Superoxide dismutase 3 (SOD3) is an extracellular enzyme with the capacity to modulate extracellular redox conditions by catalyzing the dismutation of superoxide to hydrogen peroxide. In addition to synthesis and release of this extracellular protein via the secretory pathway, several studies have shown that the protein also localizes to intracellular compartments in neutrophils and macrophages. Here we show that human macrophages release SOD3 from an intracellular compartment within 30 min following LPS stimulation. This release acutely increases the level of SOD3 on the cell surface as well as in the extracellular environment. Generation of the intracellular compartment in macrophages is supported by endocytosis of extracellular SOD3 via the LDL receptor-related protein 1 (LRP1). Using bone marrow-derived macrophages established from wild-type and SOD3^−/−^ mice, we further show that the pro-inflammatory profile established in LPS-stimulated cells is altered in the absence of SOD3, suggesting that the active release of this protein affects the inflammatory response. The internalization and acute release from stimulated macrophages indicates that SOD3 not only functions as a passive antioxidant in the extracellular environment, but also plays an active role in modulating redox signaling to support biological responses.

## Highlights

• Stimulated macrophages release SOD3 from a pre-formed intracellular compartment.
• The intracellular compartment is established by receptor-mediated endocytosis.
• Release of SOD3 from stimulated macrophages modulates the inflammatory response.
• The level of SOD3 in the extracellular space is actively controlled.

## Graphical abstract

**Figure:**
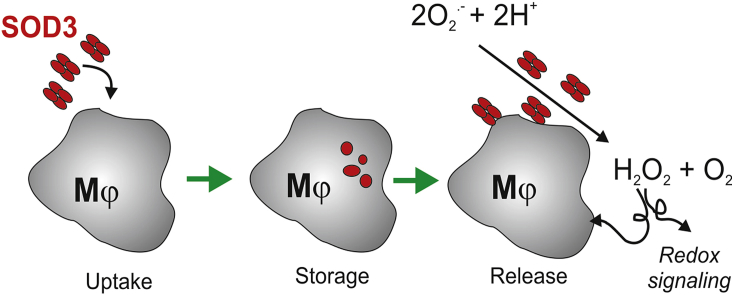

## 1 Introduction

The enzymatic activity of extracellular superoxide dismutase (EC-SOD or SOD3)^1^ supports the dismutation of superoxide to hydrogen peroxide. In addition, the protein holds the capacity to bind cell surface proteoglycans and constituents of the extracellular matrix via a positively charged *C*-terminal region (ECM-binding region) [1]. Based on these properties, SOD3 has been described as an antioxidant immobilized in the extracellular space, serving to protect cells and biomolecules against superoxide-induced damage [[2], [3], [4], [5], [6]] as well as protecting the bioactivity of nitric oxide by inhibiting the diffusion limited reaction between NO and superoxide [[7], [8], [9]]. Interestingly, in the absence of exogenous oxidative stress, mice subjected to conditional tamoxifen-induced SOD3 KO developed severe lung injury characterized by inflammatory cell infiltration and presented a significant increase in mortality [10]. This study suggests that SOD3 not only serves to protect biomolecules against superoxide-induced damage under conditions of oxidative stress, but also partakes in the control of the inflammatory response in basal conditions. The association between SOD3 and the inflammatory response has also been established in several *in vivo* models using SOD3^−/−^ mice or mice overexpressing SOD3 establishing that the protein acts as an anti-inflammatory mediator [[11], [12], [13], [14], [15], [16], [17]]. Moreover, studies have suggested a role of SOD3 in regulating transcription factor activity [18,19] as well as affecting signal transduction by regulating the activity of protein tyrosine phosphatases [20]. It is thus evident that SOD3 is not only a passive antioxidant providing extracellular protection against superoxide-mediated damage but also participates actively in regulating diverse biological functions.

Transcription of the *SOD3* gene is epigenetically regulated by both methylation of the promotor region as well as by histone acetylation/deacetylation controlling gene accessibility [[21], [22], [23], [24], [25]]. These elements are likely to explain the finding that SOD3 protein can be detected in only a limited amount of cell types including pulmonary fibroblasts and epithelial cells as well as vascular smooth muscle cells [[26], [27], [28], [29], [30]]. Indeed, the lack of SOD3 expression in human pulmonary artery endothelial cells has been shown to rely on epigenetic regulation, as the expression could be induced by demethylation of the promotor region as well as modulation of histone acetylation [22,31]. Moreover, the expression can be upregulated by cytokines including IFNγ and IL-4 whereas TNFα downregulate the expression [22,32,33]. The transcriptional regulation of SOD3 is an adaptive response that may develop over several days [32], and cannot be mobilized for an acute response.

Several studies have shown that SOD3 is present in inflammatory cells including macrophages and neutrophils [[34], [35], [36], [37]]. We have recently shown that SOD3 is only released from macrophages and neutrophils upon cellular stimulation with LPS and fMLF, respectively [36,37]. Moreover, LPS was also found to induce secretion of SOD3 from isolated astrocytes [38]. Interestingly, the secretion was not reflected by an increase in the level of SOD3 mRNA, suggesting that the release was established from a preformed population. High resolution analyses of isolated neutrophils and macrophages by electron microscopy show that SOD3 is present in intracellular vesicles [36,39]. Since SOD3 is synthesized in the secretory pathway and secreted as an extracellular protein, this finding indicates that SOD3 is internalized from the extracellular space to be stored in an intracellular compartment and only released upon stimulation. In concert, our data suggest that the stimulated release of SOD3 represents an acute response providing SOD activity to the extracellular environment.

To understand the control of SOD3 activity in the extracellular space, we have characterized the cellular distribution of SOD3 in macrophages in detail. We show that SOD3 is released within 30 min of macrophage stimulation, correlating with the kinetics of TNFα release [40]. Moreover, we show that SOD3 is mobilized from pre-formed intracellular vesicles likely established by receptor-mediated endocytosis supported by the interaction between LDL receptor-related protein 1 (LRP1) and SOD3 [41]. Analysis of the functional impact of this stimulated release was evaluated using macrophages isolated from wild-type and SOD3^−/−^ mice, and showed that the absence of SOD3 significantly increased the level of secreted pro-inflammatory cytokines and chemokines, including CXCL2 and sICAM-1. Collectively, our data show that the level of SOD3 activity in the ECM is regulated by acute cellular release and underscores that this dynamic distribution will need to be considered, when studying redox conditions at both oxidative eustress and distress.

## 2 Materials and methods

Human serum, M-CSF, 1,2-bis(2-aminophenoxy)ethane-N,N,N′,N'-tetraacetic acid tetrakis(acetoxymethyl ester) (BAPTA-AM), acutase, and ovalbumin was from Sigma. Heparin (5000 U/ml) was obtained from Leo Pharma, Denmark and complete protease inhibitor tablets was from Roche.

*Proteins:* Recombinant wild-type SOD3 and SOD3 containing a *C*-terminal c-Myc tag (SOD3-cMyc) was produced as previously described [42]. For the generation of the SOD3-cMyc expression plasmid, we used the forward primer (5′-TATACAGCTAGCATGCTGGCGCTACTGTGTTCC-3′) and a reverse primer encompassing the cMyc tag (underlined) (5′-TATGAATTCTCACAGATCCTCTTCTGAGATGAGTTTTTGTTCGGCGGCCTTGCACTCGCTCTC-3′) and cloned the product into the pIRES vector. The sequence of the obtained plasmid was verified by sequencing. mAb 5G8D4 and 7F6D9 anti-human SOD3 (GenScript) and rabbit anti SOD3 antisera (Davids Biotechnologie) were developed using recombinant human SOD3 as antigen and antibodies recovered by using protein G-Sepharose. Recombinant human and murine IFNγ was obtained from Invitrogen. Ovalbumin was from Sigma. Human receptor-associated protein (RAP) was obtained from ENZO life sciences (BML-SE552). HRP-conjugated goat anti-rabbit Ig was from DAKO. Flow cytometric analyses were performed using PE-conjugated anti-CD14 (B&D Biosciences), APC-conjugated anti-CD4 (B&D Biosciences) or APC-conjugated mAb5G8D4 anti-SOD3.

### 2.1 Cell culture

Human monocytes were isolated from buffy coats using Ficoll-paque™ PLUS (GE Healthcare). Briefly, the buffy coat material was diluted in PBS (without Mg^2+^ and Ca^2+^) containing 2 mM EDTA and the diluted material layered on top of a Ficoll-paque™ PLUS cushion and centrifuged for 30 min at 1.000×*g*. The established layer of lymphocytes and monocytes was removed and subsequently washed twice in PBS and seeded in 10 cm petri dishes in RPMI 1640 containing 2% human serum, 100 U/ml penicillin and 100 μg/ml streptomycin (hsRPMI). Monocytes were allowed to adhere for 3 h where after medium was removed and cells wash twice with PBS before receiving hsRPMI containing 20 ng/ml M-CSF. After overnight incubation, 50% of the medium was exchanged for fresh medium, and the cells allowed to differentiate into M0 macrophages and used after 7–10 days of differentiation.

### 2.2 SDS-PAGE and western blotting

SDS-PAGE analysis was performed using uniform 10% polyacrylamide gels and the glycine/2-amino-2-methyl-1,3-propanediol-HCl buffer system described previously [43]. Reducing conditions were obtained by boiling samples in the presence of 0.5% (w/v) SDS and 50 mM dithiothreitol prior to electrophoresis. For Western blotting, separated proteins were electrophoretically transferred to a polyvinylidene difluroride membrane for 1 h at 150 mA in 10 mM 3-(cyclohexylamino)-1-propane sulfonic acid (pH 11) containing 10% (v/v) ethanol [44]. The membranes were blocked with 5% (w/v) skimmed milk in 20 mM Tris-HCl (pH 7.4), 137 mM NaCl supplemented with 0.1% (v/v) Tween 20 (TBST) and SOD3 protein detected by using a rabbit anti-SOD3 antiserum. Blots were developed by ECL using peroxidase-conjugated goat anti-rabbit Ig and data acquired using an ImageQuant LAS 4000 instrument (GE Healthcare).

### 2.3 Enzyme-linked immunosorbent assays

The concentration of SOD3 in cell culture supernatants was determined by a sandwich ELISA. Microtiter wells (MaxiSorb, Nunc) were coated with a polyclonal rabbit IgG directed against SOD3 using 0.1 μg in 100 μl 50 mM carbonate/bicarbonate buffer, pH 9.6. The wells were incubated over night at 4 °C, emptied and residual binding-sites blocked for 1 h by the addition of 0.1% (w/v) BSA in TBS. Samples were diluted in TBST and a standard curve generated using purified recombinant SOD3. The wells were incubated over night at 4 °C, washed thrice in TBST, and incubated with 100 μl mAb 7F6D9 anti-SOD3 in TBST using a concentration of 1 μg/ml. Bound antibody was subsequently detected by the addition of HRP-conjugated rabbit anti-mouse antibody and wells developed by using the *O*-phenylenediamine dihydrochloride-system following the manufacturer's instructions (Sigma). Color development was assessed by absorption at 450 nm.

The detection of murine TNFα in the cell culture supernatant of stimulated bone marrow-derived macrophages was determined by using the DuoSet ELISA kit as described by the manufacturer (R&D systems).

### 2.4 Immunoprecipitation

CNBr-activated Sepharose was derivatized with mAb 5G8D4 as suggested by the manufacturer (GE Healthcare) at a concentration of 2 mg Mab/ml resin. The equilibrated resin was incubated with cell culture supernatants or cell lysates diluted in 10-fold in PBS and allowed to bind over night at 4 °C with end-over-end mixing. The resin was recovered by centrifugation, washed thrice in PBS, and bound proteins eluted by boiling the resin in the presence of SDS-PAGE sample buffer containing 0.5% SDS and 50 mM dithiothreitol.

### 2.5 RT-qPCR

Total RNA was isolated from LPS-stimulated HMDMs by using the High Pure RNA Isolation Kit (Roche) and reverse transcription subsequently performed using the SuperScript One-Step RT-PCR kit (Invitrogen). Primers used for analysis were; Sod3F, 5′-CTTCGCCTCTGCTGAAGTCT-3′); Sod3R, 5′-GGGTGTTTCGGTACAAATGG-3′; GapdhF, 5′-GCACCGTCAAGGCTGAGAAC-3′; GapdhR, 5′-ATGGTGGTGAAGACGCCAGT-3′. Relative quantitative real-time PCR used SYBR Green PCR Master Mix kit (Applied Biosystems). After pre-amplification (95 °C for 2 min), the PCRs were amplified for 40 cycles (95 °C for 15 s and 60 °C for 1 min). Each mRNA expression was normalized against GAPDH mRNA expression using the comparative cycle threshold method.

### 2.6 Release of SOD3 from human monocyte-derived macrophages (HMDM)

To evaluate the secretion of SOD3 from macrophages, HMDMs were seeded in 6 well plates (5 × 10^4^ cells/cm^2^) and allowed to adhere overnight. Subsequently, cells were washed in PBS, stimulated in 2 ml hsRPMI containing 100 ng/ml LPS and the cell culture supernatant recovered at the indicated time-points. To evaluate the involvement of exocytosis, cells were washed in PBS and pre-incubated for 15 min in PBS containing the indicated concentrations of BAPTA-AM, which inhibits vesicular exocytosis by the chelation of intracellular calcium. The secretion of SOD3 was subsequently stimulated by the addition of 100 ng/ml LPS for 1 h.

### 2.7 Cellular uptake of SOD3

Human macrophages were seeded in 6 well plates (5 × 10^4^ cells/cm^2^) and pulsed with SOD3-cMyc (500 ng/ml) or ovalbumin (500 ng/ml) in 2 ml fcsRPMI for 90 min. Cells were subsequently washed in PBS containing 50 U/ml heparin and lysed at the indicated time points by the addition of ice-cold 50 mM Tris–HCl, 150 mM NaCl, 2 mM EDTA, 1% Triton X-100, 20 mM *N*-ethylmaleimide, pH 7.4, supplemented with complete protease inhibitors (lysis buffer). Cellular uptake of SOD3-cMyc was likewise evaluated in the presence of heparin or receptor associated protein (RAP) as indicated.

### 2.8 Flow cytometry

Human monocyte-derived macrophages were stimulated by using 100 ng/ml LPS in hsRPMI for the indicated time points. Cells were recovered by using acutase, washed twice in hsRPMI and maintained in hsRPMI at 10^6^ cells/ml. To allow for antibody labelling, 0.5 × 10^6^ cells (500 μl) were removed and incubated with PE-conjugated anti-CD14, APC-conjugated anti-CD4 or APC-conjugated 5G8D4 anti-SOD3. Moreover, we included an isotype control for 5G8D4 (APC-conjungated IgG_1_) as a negative control. All incubations were performed at 23 °C and in the dark. Flow cytometric analysis was performed using a LSR Fortessa cell analyzer (B&D Biosciences) using a 640 nm laser to excite APC (emitted light collected in a 670/30 band pass filter), and a 561 nm laser to excite PE (emitted light collected in a 586/15 band pass filter). More than 10,000 cells were counted for each sample. The obtained data were processed using the FlowJo software (V.9.6.2, TreeStar Inc, Ashland, OR).

### 2.9 Confocal microscopy

Human monocytes were purified as above, except that Ficoll-paque™ PLUS separation was followed by CD11b-immunomagnetic isolation according to recommendations of the manufacturer (Milteny). Monocytes were cultured as above and differentiated for 7 days with M-CSF (20 ng/ml) before use. To assess exocytosis and surface disposition of SOD3, macrophages were stimulated with 100 ng/ml LPS for 1 h before wash in ice-cold HBSS. Subsequently, 2 μg/ml anti-SOD3 mAb 7F6D9 was added together with 2 μg/ml Alexa633-conjugated cholera toxin B-subunit (CTB) for 30 min on ice to detect SOD3 on the surface, before wash and Alexa488-conjugated secondary goat-anti mouse antibodies. After a final wash, cells were fixed in paraformaldehyde for observation. Immunofluorescence was performed on paraformaldehyde fixed macrophages according to standard procedures using permeabilization with 0.2% saponin. In some wells cells were allowed to endocytose DiI-conjugated Acetylated-LDL (AcLDL) and Alexa633-conjugated CTB for 2 h before fixation. The following primary antibodies were used: anti-SOD3 polyclonal rabbit antibodies ESC1 and ESC2, *anti*-AP3δ mAb SA4 and anti-LAMP1 polyclonal mAb H4A3 (both Iowa Hybridoma Gene Bank), followed by Alexa488-conjugated goat-anti mouse antibodies or Alexa568-conjugated goat-anti mouse antibodies, as specified. Cells were counter stained with Alexa633-conjugated phalloidin to reveal cell shape. Slides were examined with a Zeiss LSM 510 confocal laser scanning microscope using a *C*-Apochromat 633, 1.2 water immersion objective (Carl Zeiss). Images were collected and saved as a 1024 × 1024-pixel image at 8-bit resolution before import into Adobe Photoshop (Adobe Systems) for compilation.

### 2.10 Mouse cytokine array analysis

Murine bone marrow-derived macrophages were established from femurs and pelvises recovered from wild-type and SOD3^−/−^ mice as previously described [37]. Isolated and differentiated cells were seeded in 6 well plates (0.5 × 10^6^ cells) and stimulated for 16 h by 10 ng/ml murine IFNγ in RPMI supplemented with 10% FCS, 100 U/ml penicillin and 100 μg/ml streptomycin. To stimulate SOD3 release, 100 ng/ml LPS was added to the medium and cell culture supernatants (2 ml) were removed after 16 h. Equal volumes of supernatants representing wild-type or SOD3^−/−^ mice were pooled and used to probe the Mouse cytokine array panel A as described by the manufacturer (R&D Biosystems). The intensities of the spots developed on the membranes were determined by using the ImageJ software and relative intensity adjusted on basis of the 6 reference points. The normalized pixel intensity of the individual cytokines was subsequently determined.

## 3 Results

### 3.1 The fast release of SOD3 from macrophages reflects protein secretion

**Fig. 1:**
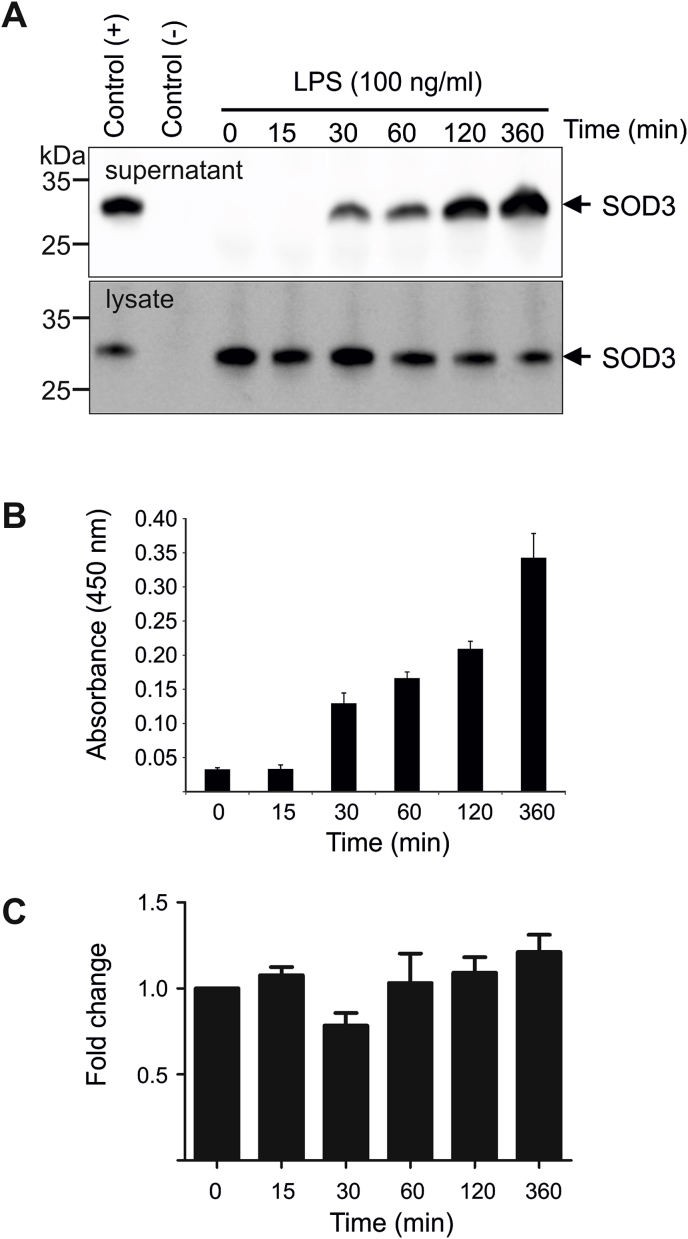
SOD3 is released from a preformed pool. HMDMs were stimulated by the addition of LPS and the supernatant collected at the indicated time points. The supernatant and corresponding cell lysates were analyzed by (A) immunoprecipitation/Western blotting. As a control of immunoprecipitation, we performed parallel analysis in the absence (−) or presence (+) of purified recombinant SOD3. Additionally, the presence of SOD3 in the supernatant was evaluated by ELISA (B). (C) The transcriptional level of *SOD3* upon LPS stimulation was evaluated by RT-qPCR and presented relatively to the level in the absence of LPS (t = 0 min). This analysis shows that the release of SOD3 from macrophages is relative fast and does not reflect a transcriptional upregulation. Error bars in panel B and C represents mean ± SD (*n* = 3).

**Fig. 2:**
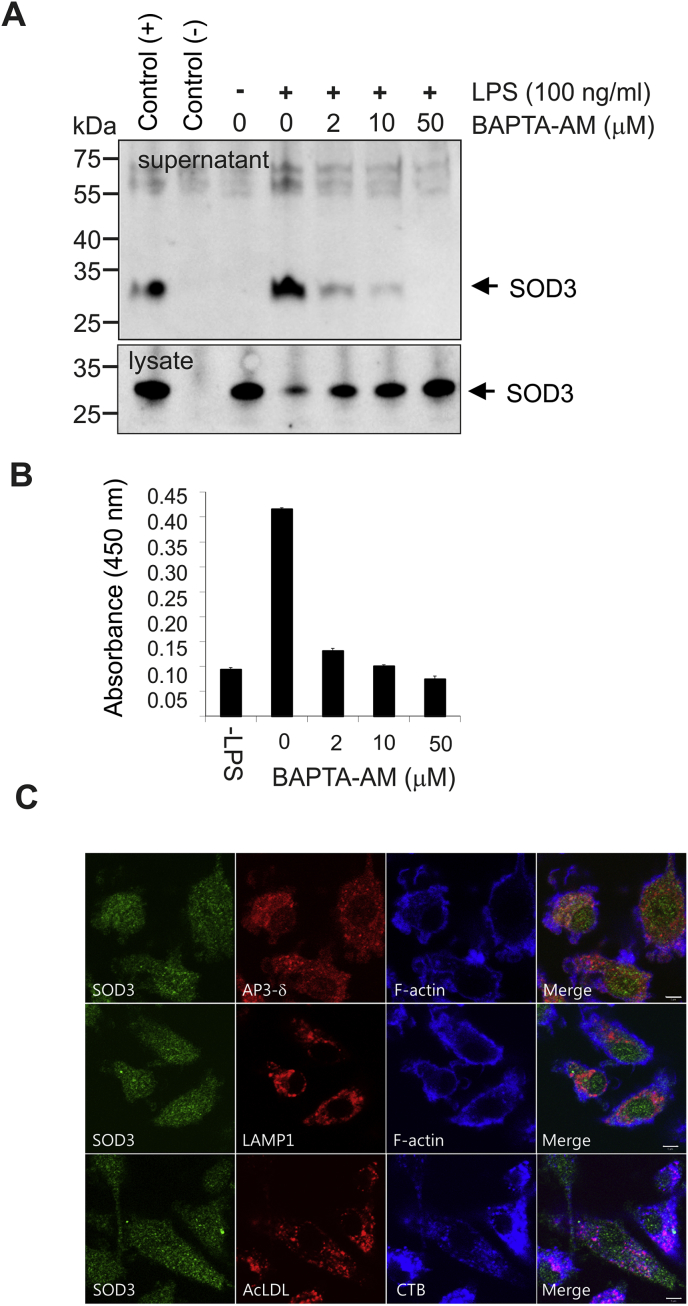
LPS-stimulation mobilize SOD3-containing intracellular vesicles. (A) HMDMs were pre-incubated with BAPTA-AM at the indicated concentrations and SOD3 release subsequently stimulated by the addition of LPS (100 ng/ml). Cell culture supernatants and lysates were analyzed for the presence of SOD3 by immunoprecipitation and SDS-PAGE/Western blotting. Control for immunoprecipitation is indicated on the left. (B) The level of SOD3 in the cell culture supernatant was likewise determined by using ELISA. (C) Immunofluorescence of unstimulated HDMDs shows an intracellular localization of SOD3 in small vesicles, that does not overlap with localization of lysosomal markers AP3δ, LAMP1 or DiI-conjugated Ac-LDL. Images are representative of three separate experiments. These data show that SOD3 is mobilized from stimulated macrophages by exocytosis of intracellular vesicles.

We have previously shown that SOD3 is actively released from LPS-stimulated murine bone marrow-derived macrophages [37]. To further evaluate the dynamics of this release, we stimulated HMDMs with LPS and collected cell culture supernatants at increasing time points. Analyses by Western blotting (Fig. 1A) and ELISA (Fig. 1B) show that SOD3 can be detected in the cell culture medium after 30 min of stimulation and that the released level continues to increase within 6 h. Correspondingly, the level of SOD3 in the cell lysates decreased (Fig. 1A). Determination of transcriptional level of SOD3 by quantitative RT-PCR shows that the level of SOD3 mRNA is not significantly affected within 6 h, suggesting that release of SOD3 does not reflect increased transcriptional activity (Fig. 1C). These data show that the SOD3 protein released from macrophages is pre-formed and mobilized from a cellular compartment. To support this conclusion, we pre-incubated macrophages with BAPTA-AM, which is cell permeable chelator capable of inhibiting vesicular exocytosis by the chelation of intracellular calcium. Analysis of cell culture supernatant collected from cells stimulated with LPS clearly shows that the presence of BAPTA-AM inhibits the release of SOD3, with complete inhibition observed by using 50 μM BAPTA-AM (Fig. 2A and B). The inhibition of release was reflected by the retention of SOD3 in the cell lysates, as evident by Western blot analysis (Fig. 2A). In agreement with these results, we found by immunofluorescence analysis that macrophages contain an intracellular pool of SOD3 in small vesicles (Fig. 2C). We addressed whether this pool of SOD3 could potentially be contained in so-called lysosome related organelles (LRO's), however we found little colocalization with lysosomal marker LAMP1 or the lysosomally directed endocytic probe acetylated-LDL (Ac-LDL), and neither did SOD3 staining overlap with AP3δ, which has been implicated in sorting to LRO's. Endocytosed CTB, which transits through endosomes and accumulates in the Golgi, neither showed any appreciable colocalization with SOD3. In concert, these data show that LPS stimulation of HMDMs mobilize intracellular SOD3-containing vesicles supporting the release of the protein into the extracellular environment.

### 3.2 Cell surface-associated SOD3 is increased in LPS-stimulated macrophages

**Fig. 3:**
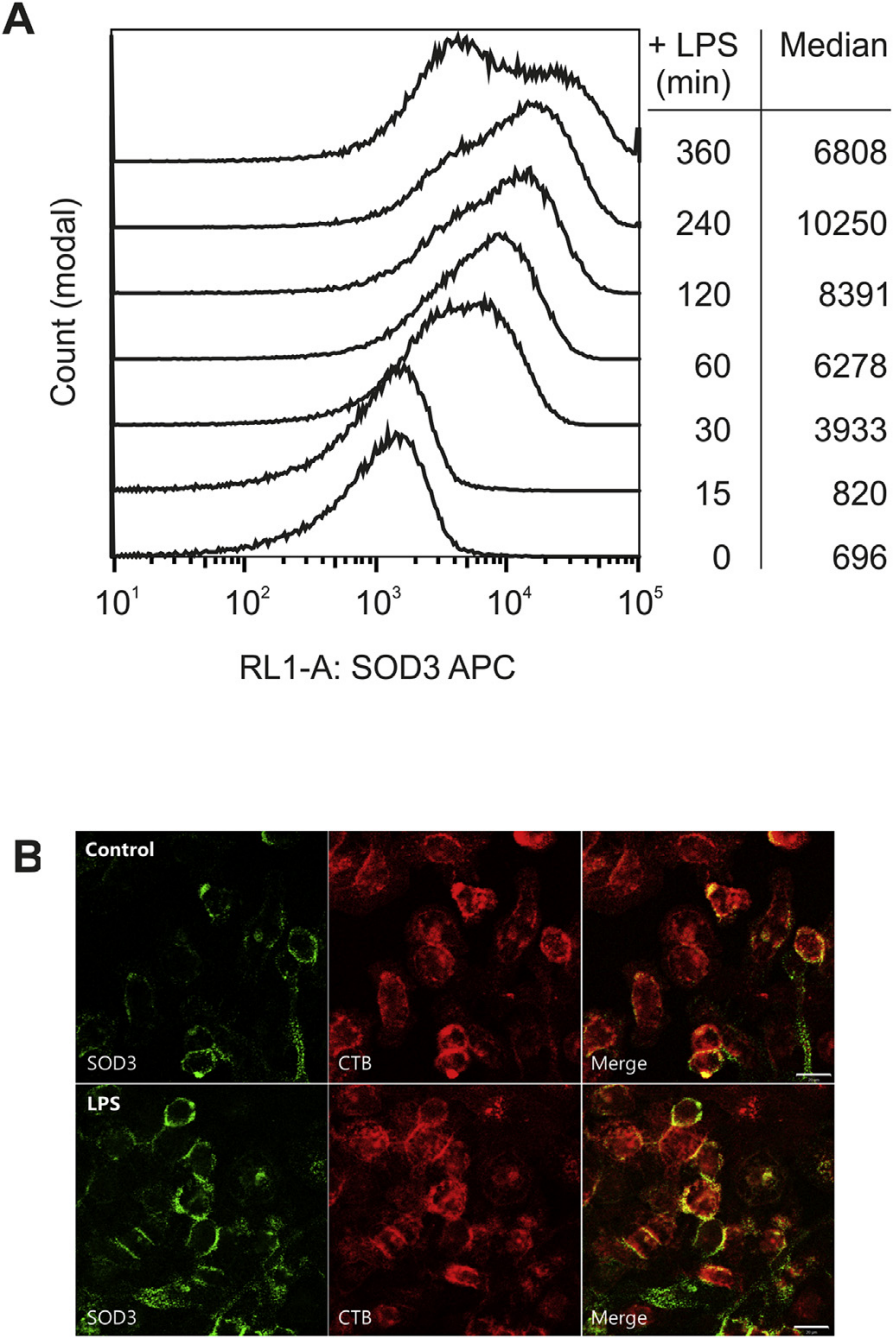
Cell surface-associated SOD3 in increased upon stimulation. (A) The level of cell surface-associated SOD3 on resting and LPS-stimulated HMDMs was evaluated by using flow cytometry. Stimulated cells were fixed after the indicated time points and surface associated SOD3 detected by using an APC-conjugated mAb 5G8D4. Cells were gated to select live and monodisperse HMDMs (Fig. S1). The analysis was repeated three times with cells obtained from separate donors producing similar results. (B) Cell surface-associated SOD3 was evaluated by confocal microscopy. Cell surface staining of SOD3 and cholera toxin B-subunit (lipid rafts) on live HDMDs treated or not with LPS shows a substantially increased localization of SOD3 to the surface of LPS-treated cells. These analyses show that the level of cell surface-associated SOD3 is increased by LPS stimulation.

**Fig. S1:**
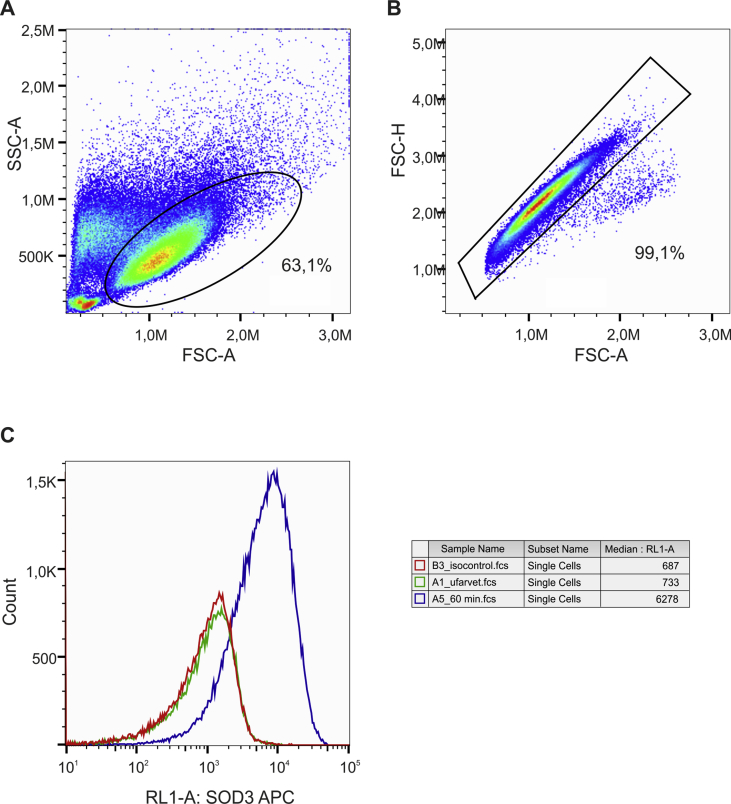
Gating strategy for HMDMs. (A) Live cells were gated from dead cells and debris by forward-scatter area (FSC-A) and side-scatter area (SSC-A). (B) Monodisperse cells were subsequently selected on basis on FSC-A and forward-scatter height (FSC–H). (C) To validate the specificity of the staining procedure cells were analyzed unstained (green) or by using and APC-conjugated isotype control (IgG_1_) (red). Cells stimulated by LPS for 60 min are included as reference (blue).

Since the stimulation of HMDMs by LPS appears to mobilize intracellular stores of SOD3, we investigated if vesicular exocytosis also supported an increase of cell surface-associated SOD3 by flow cytometry. The differentiation of the cells was verified by using CD4 and CD14 antibodies and viable and monodisperse HMDMs were gated using forward and side scatter channels (Fig. S1). The specificity of the analysis confirmed by staining of cells using an isotype control antibody, which showed no intensity above background (Fig. S1). To allow for the analysis of surface-associated SOD3 only, cells were not permeabilized prior to the analysis. Analysis of stimulated cells showed that the level of cell surface-associated SOD3 increased within the first hours of stimulation as evident by increasing median fluorescent values (Fig. 3). After 6 h of stimulation, the median value decreased, indicating the level of surface-associated SOD3 is reduced following an initial burst. Based on the >10-fold increase of the median value between the initial state (696) and at 4 h (10250), these data suggest that the level of SOD3 on the cell surface is increased dramatically. To obtain a visual correlate, HMDMs were stimulated with LPS and the localization of SOD3 on the cell surface was detected by anti-SOD3 antibodies on living cells for confocal microscopical analysis. LPS afforded a clearly increased complement of SOD3 on the cell surface (Fig. 3B). Moreover, LPS-stimulation increased the association with lipid rafts (CTB) as previously described [37]. In concert, LPS stimulation of HMDMs markedly affects the cellular distribution of SOD3 increasing the level of both cell surface-associated as well as protein released into the extracellular environment.

### 3.3 Internalization and release of exogenous SOD3

**Fig. 4:**
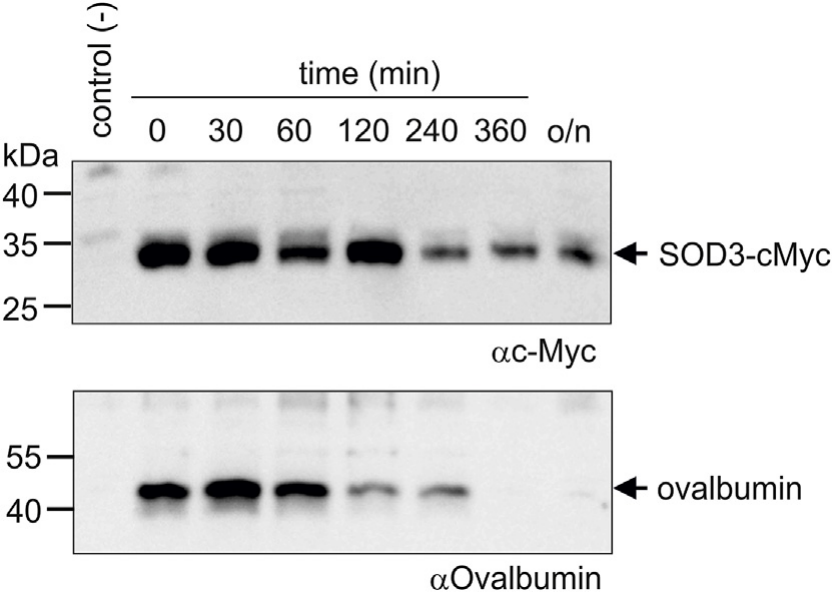
SOD3 is internalized and stored intracellularly. (A) HMDMs were added medium containing ovalbumin or cMyc-tagged SOD3 and incubated for 90 min. The medium was subsequently removed, the cells washed in the presence of heparin (50 U/ml) and cells subsequently lysed at the indicated time points. The recovered lysates were analyzed for the presence of ovalbumin and SOD3-cMyc by SDS-PAGE/Western blotting. The analysis was performed three times. This analysis shows that internalized SOD3-cMyc is maintained within the cell whereas ovalbumin is degraded.

**Fig. 5:**
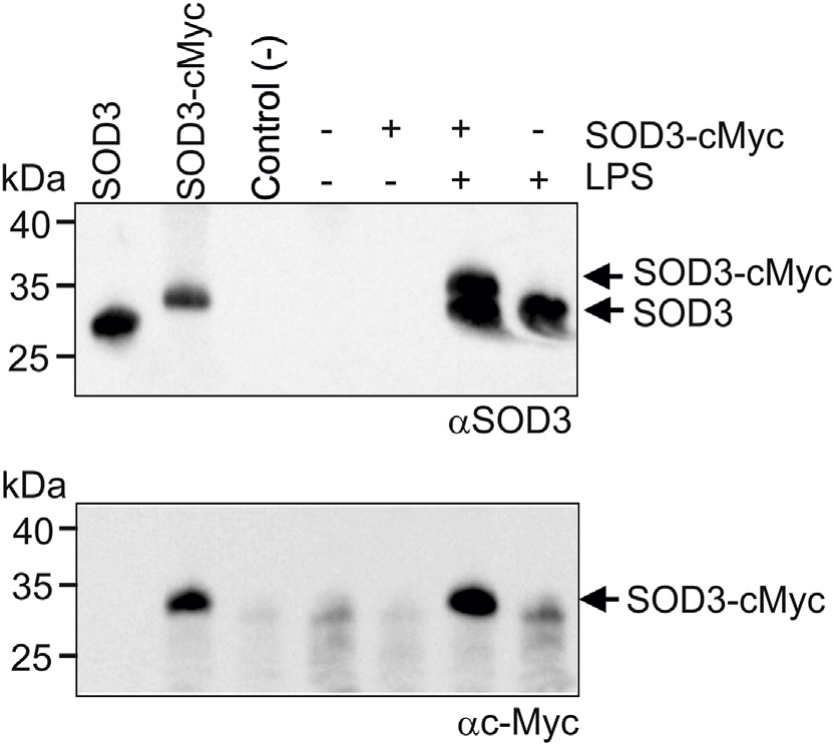
Internalized SOD3 is released from stimulated HMDMs. Cells were cultured in the absence or presence of SOD3-cMyc for 90 min to allow for internalization. Cells were washed and added buffer or stimulated by the addition of LPS for 1 h as indicated. Cell culture supernatants were collected and analyzed by immunoprecipitation and SDS-PAGE/Western blotting. Two separate membranes were developed using an anti-SOD3 antiserum and Mab 9E10 *anti*-cMyc. Purified SOD3 and SOD3-cMyc was included as controls on the left. This analysis shows that internalized SOD3 is released from HMDMs upon LPS stimulation.

Since SOD3 is an extracellular glycoprotein without any well-defined tags for cellular sorting prior to secretion, it is likely that the establishment of intracellular vesicles is mediated by endocytosis of secreted protein. Indeed, SOD3 has previously be reported to be internalized by endothelial cells and fibroblasts [[45], [46], [47]], as well as by receptor-mediated endocytosis by hepatocytes [41]. Moreover, we have recently shown that SOD3 is present in secretory vesicles of neutrophils, which are established by endocytosis [36]. To evaluate endocytosis, we incubated HMDMs in the presence of recombinant SOD3 encompassing a *C*-terminal c-Myc tag (SOD3-cMyc) allowing us to distinguish between endogenous and exogenous SOD3 by using an antibody directed against the cMyc-tag. In parallel, we incubated cells with ovalbumin serving as a marker of endocytosis and intracellular degradation. After a pulse of 90 min, cells were washed in PBS containing heparin to remove cell surface associated SOD3-cMyc and maintained in medium for the indicated time points (Fig. 4). Analysis of cell culture lysates by Western blotting shows that SOD3-cMyc is internalized and can be detected within the macrophage after overnight incubation. Ovalbumin was also internalized but could not be detected after 6 h, indicating that the protein is degraded (Fig. 4). These data show that SOD3 is internalized by macrophages and that a fraction of the protein is likely stored in an intracellular compartment. To investigate if internalized SOD3-cMyc could be released from this intracellular compartment, we pulsed HMDMs with SOD3-cMyc, washed the cells in the presence of heparin and stimulated them with LPS (Fig. 5). Correlating with data presented in Fig. 1A, SOD3 could not be detected in the supernatant in the absence of LPS stimulation whereas the presence of LPS stimulated the release, as evident by Western blotting using an anti-SOD3 antiserum (Fig. 5, upper panel). In addition, we could also detect SOD3-cMyc protein in the culture supernatant of LPS-stimulated cells pulsed with SOD3-cMyc protein using both an SOD3-directed antiserum (upper panel) as well as a specific *anti*-cMyc antibody (Fig. 5, lower panel). These data show that SOD3 is internalized, stored, and subsequently released upon LPS stimulation.

### 3.4 SOD3 is internalized by receptor-mediated endocytosis

**Fig. 6:**
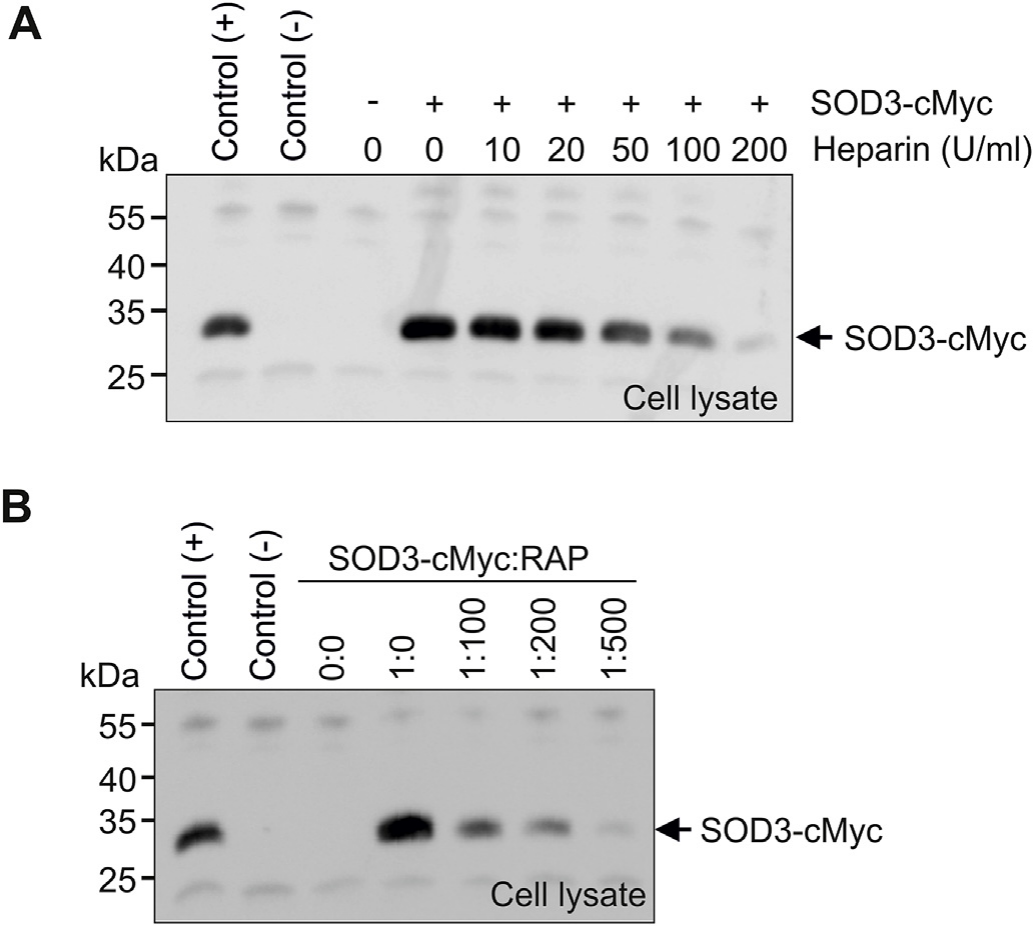
SOD3 is internalized by receptor-mediated endocytosis. (A) HMDMs were incubated with SOD3-cMyc (500 ng/ml) in the presence of increasing amounts of heparin as indicated for 90 min. Cells were subsequently washed, lysed and the recovered lysate analyzed by immunoprecipitation and SDS-PAGE/Western blotting. The uptake of SOD3 was evaluated by using the mAb 9E10 *anti*-cMyc. (B) The involvement of LRP1 in internalization was evaluated by incubating SOD3-cMyc in the presence of increasing molar ratios of RAP, an inhibitor of LRP1-ligand interactions. The cells were incubated in 90 min and the lysates analyzed as in (A). The experiments were repeated three times with similar results. This analysis shows that SOD3 is internalized into HMDMs via LRP1 interaction.

To investigate the basis of SOD3 uptake in human macrophages, we incubated HMDMs with SOD3-cMyc in the presence of increasing amounts of heparin to compete for cell surface binding of SOD3-cMyc via the ECM-binding region. Analysis of cell lysates by Western blotting shows that heparin dose-dependently inhibits the internalization of SOD3-cMyc (Fig. 6A), suggesting that the interaction with cell surface heparan sulfate proteoglycans is essential for cellular uptake. We have previously shown that SOD3 interacts with the cell surface receptor LRP1 (CD91) [41]. Since the binding to cell surface heparan sulfate proteoglycans is important for the internalization of a number of LRP1 ligands, we investigated if LRP1 could support SOD3 internalization. To investigate this, we used the receptor-associated protein (RAP), which is a universal inhibitor of LRP1-ligand interactions [48]. Analysis of lysates obtained from HMDMs incubated with SOD3-cMyc in the presence of increasing levels of RAP, showed that the internalization was inhibited by RAP (Fig. 6B). Collectively, these data show that the interaction between LRP1 and SOD3 mediated by heparan sulfate proteoglycans supports the internalization of SOD3 in HMDMs.

### 3.5 Secretion of SOD3 affects the macrophage cytokine response

**Fig. 7:**
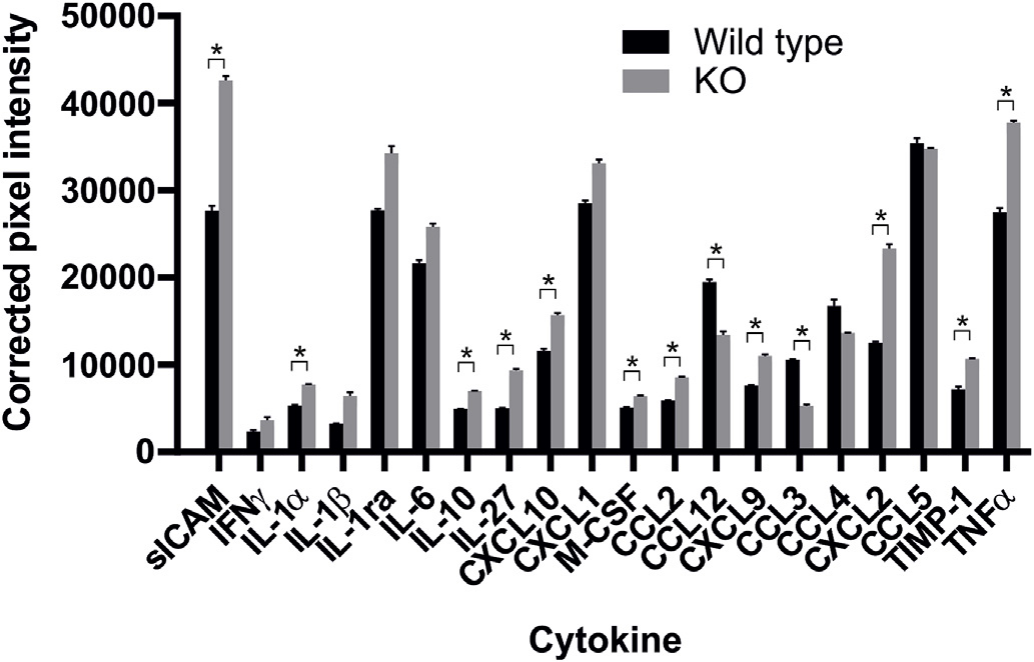
SOD3 modulates the pro-inflammatory cytokine profile of LPS-stimulated macrophages. Bone marrow-derived macrophages were established from wild-type and KO mice and stimulated by IFNγ and LPS for 16 h. Cell culture supernatants were collected and the pro-inflammatory cytokine profile was established by using the Mouse cytokine array panel A (R&D systems). Cell culture supernatants were pooled from wild-type mice (*n* = 3) or SOD3 KO mice (*n* = 3) and used to probe the membrane. The intensity of the developed spots was evaluated as described by the manufacturer by using ImageJ software and presented as corrected pixel intensity with error bars indicating mean ± SD. Data were analyzed by Student's t-test and *p* < 0.05 indicated (*).

**Fig. S2:**
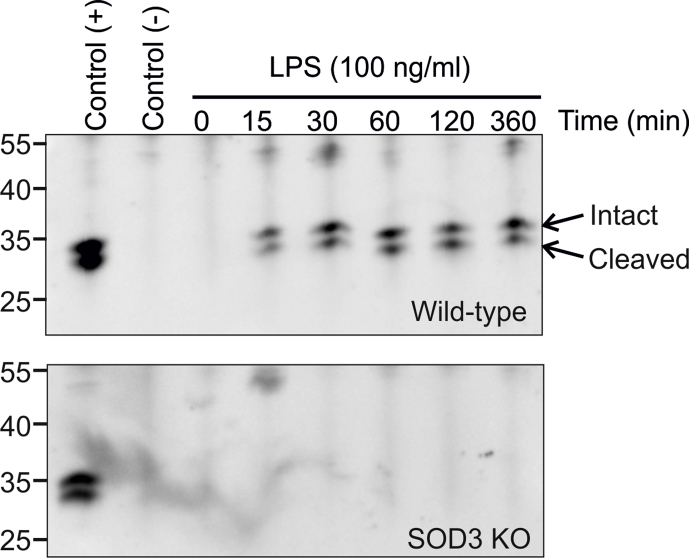
Murine SOD3 is secreted from macrophages derived from wild-type animals. BMMs differentiated from wild-type and SOD3^−/−^ animals were incubated in culture medium containing 100 ng/ml LPS. Following the indicated time-points, culture supernatant was collected and immunoprecipitated using anti-mouse SOD3 antiserum (S19; Santa Cruz). Precipitates were analyzed by SDS-PAGE/Western blotting. The analysis shows that cells derived from wild-type animals secrete detectable SOD3 within 15 min of incubation (upper panel), whereas no SOD3 could be detected in SOD3^−/−^ cells (lower panel).

**Fig. S3:**
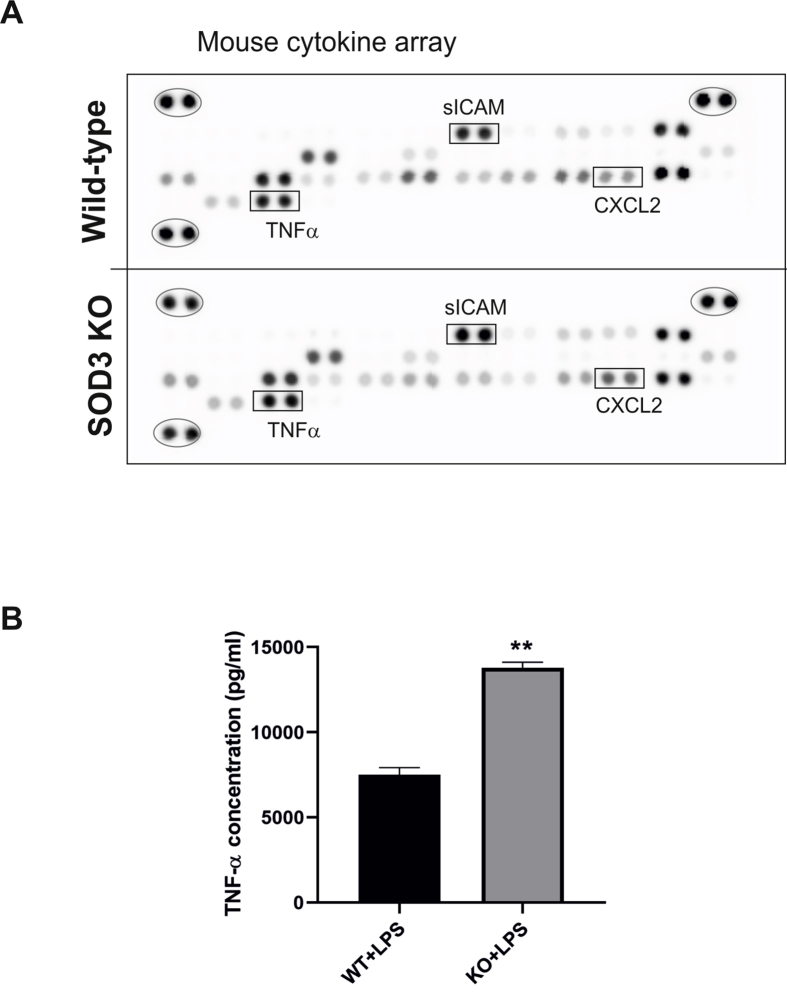
The cytokine response from BMMs is affected by SOD3. (A) Cell culture supernatants from LPS-stimulated BMMs derived from wild-type (*n* = 3) or SOD3^−/−^ mice (*n* = 3) were pooled and used to probe a mouse cytokine panel. The intensity of the dots was evaluated by using ImageJ software and normalized by use of reference points (ovals). The position of sICAM1, CXCL2 and TNFα is indicated (squares). (B) The level of secreted TNFα was likewise evaluated by using a TNFα-specific ELISA assay (DuoSet, R&D systems). Levels were evaluated by Student's t-test and *p* < 0.05 indicated (**).

One important effector function of activated macrophages is the secretion of cytokines. To evaluate if the temporal secretion of SOD3 from LPS-stimulated macrophages affected the profile of secreted cytokines, we established bone marrow-derived macrophages (BMM) from both wild-type and SOD3^−/−^ mice and stimulated a pro-inflammatory response by the addition of IFNγ and LPS. Analysis by Western blotting showed that indeed SOD3 was quickly released from BMMs isolated from wild-type animals (Fig. S2), with a kinetic profile comparable to that of HMDMs (Fig. 1A). As expected, no SOD3 was released from BMMs established from SOD3^−/−^ mice (Fig. S2). Supernatants collected from BMMs representing wild-type or SOD3^−/−^ mice were pooled and used to probe a mouse cytokine array panel (Fig. S3A). This analysis showed that the release of several cytokines were significantly increased in the absence of SOD3, including TNFα (Fig. 7). To validate the findings obtained by the array, we analyzed the collected supernatants by using a TNFα-specific ELISA. This analysis confirmed that indeed the level of this cytokine was significantly elevated in supernatants of BMMs derived from SOD3^−/−^ mice (Fig. S3B). Interestingly, in addition to TNFα upregulation, the chemokine CXCL2 (MIP2α) was also found to be significantly upregulated in the absence of SOD3, correlating well with the observation that overexpression of SOD3 in mice significantly reduce the level these two cytokines in a model of LPS-induced pulmonary inflammation [11]. In conclusion, the absence of SOD3 affects the profile of cytokines produced by isolated macrophages and indicates that stimulus-induced release of SOD3 indeed affects inflammatory response established by the macrophage. This notion correlates well with the body of evidence showing that SOD3 acts as an anti-inflammatory protein in a number of *in vivo* models [11,12,14,16].

## 4 Discussion

By virtue of its enzymatic activity, SOD3 has the capacity to remove superoxide generated in the extracellular space, and hence protect constituents in this compartment against oxidative modification and degradation [[2], [3], [4], [5], [6]]. However, the activity of SOD3 can also be appreciated as a source of H_2_O_2_ in the extracellular environment for potential autocrine or paracrine redox signaling. As H_2_O_2_ plays a central role in redox signaling, it is highly plausible that SOD3 indeed will affect redox-regulated processes and hence function as both an antioxidant as well as a prooxidant in the extracellular space [49]. The impact of this notion is becoming increasingly relevant, as it has been established that H_2_O_2_ crosses biological membranes not only by passive diffusion, but also by active transport supported by peroxiporins (aquaporins) [[50], [51], [52]] and that this transport indeed impacts on cellular biology [53]. This implies that H_2_O_2_ generated in the extracellular space may affect the redox status of intracellular targets, and it is consequently essential to understand how the activity of SOD3 in the extracellular space is regulated.

We have previously shown that SOD3 is released into the extracellular environment from LPS-stimulated macrophages [37]. Now we show that this release is supported by the mobilization of an intracellular compartment within 30 min after stimulation. This fast release correlates with that observed for TNFα [40] and indicates that the release of SOD3 is important in the early response of the macrophage. The molecular basis of this release is not clear, as the protein collected in the supernatant appears to be intact and hence withholds the ECM-binding region (Fig. 1A) [37]. However, as BAPTA-AM inhibits this process (Fig. 2) it is evident that exocytosis is essential. It could be speculated, that the acute release of SOD3 by exocytosis would saturate the cell surface and hence establish an equilibrium between SOD3 in the culture supernatant and cell-surface associated protein. However, flow cytometric analysis shows that the cell surface is not saturated at 30 min (Fig. 3), and hence cell surface saturation is likely not the basis for the observed release. It may also be hypothesized, that the activity of, e.g. heparanase, would release SOD3 from the cell surface by cleaving heparan sulfate chains on cell surface proteoglycans; a process known to release pro-inflammatory cytokines including TNFα and IL-1β [54,55]. Our previous findings show the level of SOD3 on the macrophage cell surface was unaffected by LPS stimulation as evaluated after 18 h [37]. Here we show, that within 4 h the level of surface-associated SOD3 increase and levels off when evaluated at 6 h (Fig. 3). These findings suggest that exocytosis of SOD3-containing vesicles, supports an initial burst of SOD activity at the cell surface. To what extent this correlates spatially and temporally with the agonist-regulated trafficking of NADPH oxidase 2 (NOX2)-containing secretory vesicles to the cell surface is at present unknown [56]. With the active transport of H_2_O_2_ by peroxiporins, it is thus possible that the oxidative burst will provide elevated levels of H_2_O_2_ in the intracellular space to affect the cellular response, as observed in endothelial cells [20]. Despite the lack of mechanistic insight, it is clear that the release of SOD3 from the macrophage could provide a basis for intercellular communication mediated by SOD activity.

**Fig. 8:**
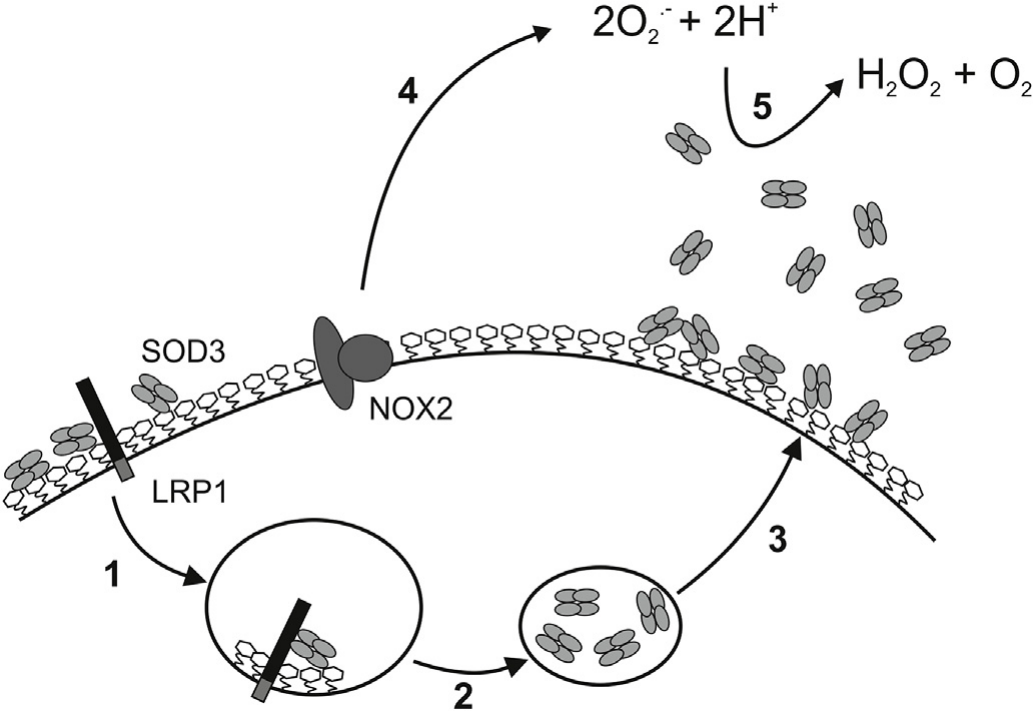
Schematic showing the cellular cycle of SOD3 in macrophages. The secreted protein binds to glycosaminoglycans on the cell surface and is internalized via the interaction with LRP1 (1). The internalized SOD3 is sorted and stored in intracellular vesicles of unknown identity (2). Upon stimulation, the macrophage has the capacity to mobilize SOD3 from these vesicles to allow for an acute increase at the cell surface as well as in the surrounding tissue (3). The concomitant expression of NOX2 activity supports the generation of superoxide (O_2_^.-^) (4). The increased SOD3 activity in the extracellular space has the capacity to modulate the redox environment and affect endocrine and paracrine redox-dependent signaling by generating H_2_O_2_ as a secondary messenger (5).

LRP1 is an endocytic receptor and highly expressed by macrophages [57]. The receptor has the capacity to internalize extracellular ligands and support intracellular sorting by directing ligands to, e.g., the lysosomal compartment for degradation or to recycling endosomes for subsequent release. We show that the uptake of SOD3 into HMDMs is mediated by the interaction with LRP1. Supported by flow cytometric analysis of un-stimulated cells, we suggest that SOD3 synthesized in macrophages is secreted from the cell and immobilized on the cell surface via the interaction with heparan sulfate proteoglycans (Fig. 8). This provides a basis for the interaction with LRP1 and allows for internalization and subsequent sorting into a subcellular compartment detected by confocal microscopy (Fig. 2) and electron microscopy [39] (Fig. 8). The identity of this compartment is not yet been established. However, intracellular SOD3 did not to any appreciable degree colocalize with lysosomal markers, and thus SOD3 is probably not contained in LROs, which in macrophages constitute a well known exocytic compartment. Further analysis will be required to reveal the identity of the SOD3-containing organelles.

Endocytosis of SOD3 will maintain a steady-state level of SOD3 on the macrophage cell surface to support redox homeostasis. LPS-stimulation will subsequently mobilize this compartment and allow for the release of SOD3 into the extracellular space whereby an acute increase in SOD activity is established (Fig. 8). Since the transcriptional regulation of SOD3 expression is slow, the stimulus-induced release of a preformed compartment provides the macrophage with an immediate redox-modulating factor. Interestingly, NOX2 is contained in a similar agonist-regulated non-LRO compartment [56]. The assembly of the NOX2 complex provides a similar acute redox modulating response, by recruiting cytosolic factors to the membrane-associated gp91^phox^/p22^phox^ complex [58].

By using BMMs isolated from wild-type and SOD3^−/−^ mice, we show that the stimulus-induced release of SOD3 attenuates the secretion of a number of pro-inflammatory cytokines, demonstrating that the release induce a functional impact on macrophage biology (Fig. 7). We find that the level of the chemokines CXCL1 and CXCL2 secreted from macrophages is increased in the absence of SOD3, although the former is not statistically significant (Fig. 7). This finding correlates with a previous study showing that exogenous SOD3 present in culture medium of an LPS-stimulated macrophage cell line attenuates the level of secreted CXCL2 [11]. Since CXCL1 and CXCL2 are chemokines central to neutrophil trafficking [59], these data support the findings of several *in vivo* models of inflammation presenting an augmented level of neutrophil recruitment in SOD3^−/−^ animals [3,10,16,60]. Additionally, the level of sICAM-1 released from macrophages was also found to increase in the absence of SOD3. This association has previously been observed in patients presenting active rheumatoid arthritis, establishing that the level of sICAM in serum correlates negatively with the level of SOD3 [61]. Interestingly, the level of TNFα and CXCL2 released from rat alveolar macrophages was found to increase when cells were stimulated with sICAM [62]. The finding that SOD3^−/−^ macrophages release relative higher levels of sICAM therefore substantiates the augmented levels of both TNFα and CXCL2 secreted from these cells. Conclusively, the modulated cytokine profile shows that the induced secretion of SOD3 from an intracellular compartment will affect the biological response of the macrophage, and therefore validates the notion, that SOD3 has the capacity to affect redox-regulated events.

We show that the level of SOD3 in the extracellular space is regulated by cellular uptake and release in macrophages. The induced release allows the macrophage to acutely modulate the redox conditions in the extracellular space and suggests that SOD3 plays an important role in the early phase of macrophage activation and the inflammatory response. Further studies are warranted to describe mechanisms involved in intracellular storage and release of SOD3. Our study adds to the current knowledge suggesting that SOD3 must be considered as an active player during the inflammatory response.

## Author contributions

Conceived and designed the experiments: S.V.P., F.V. Performed the experiments: L.H., E.D.Z., U.G.L. Analyzed the data: S.V.P., F.V., L.H., E.D.Z. Wrote the paper: S.V.P., F.V. All authors reviewed the manuscript and provided editorial input.

## Abbreviations

BMM: Bone marrow-derived macrophage
CTB: Cholera toxin B-subunit
ECM: Extracellular matrix
HMDM: Human monocyte-derived macrophage
sICAM1: Soluble intercellular adhesion molecule 1
LRP1: LDL receptor-related protein 1
NOX2: NADPH oxidase 2
RAP: Receptor-associated protein
SOD3: Superoxide dismutase 3

## Footnotes

1 We suggest that the nomenclature of superoxide dismutase isozymes in mammals should be SOD1, SOD2 and SOD3, representing Cu/Zn-SOD, Mn-SOD and EC-SOD. This nomenclature is increasingly used in the literature. Moreover, the latter notion mix the type of associated metals (Cu/Zn-SOD, Mn-SOD) with spatial localization (EC-SOD), which makes this nomenclature inconsistent. In addition, although SOD3 is processed through the secretory pathway, our data and data by others show that the distribution of SOD3 may be more complex and not only associated with the extracellular space.
